# Hybrid Blockchain Platforms for the Internet of Things (IoT): A Systematic Literature Review

**DOI:** 10.3390/s22041304

**Published:** 2022-02-09

**Authors:** Ahmed Alkhateeb, Cagatay Catal, Gorkem Kar, Alok Mishra

**Affiliations:** 1 Department of Computer Engineering, Bahcesehir University, Istanbul 34353, Turkey; mohamed.alkhateeb@bahcesehir.edu.tr (A.A.); gorkem.kar@eng.bau.edu.tr (G.K.); 2 Department of Computer Science and Engineering, Qatar University, Doha 2713, Qatar; ccatal@qu.edu.qa; 3 Informatics and Digitalization Group, Molde University College—Specialized University in Logistics, 6410 Molde, Norway; 4 Software Engineering Department, Atilim University, Ankara 06830, Turkey

**Keywords:** Internet of Things, blockchain, cloud computing, integration, hybrid blockchains, systematic literature review

## Abstract

In recent years, research into blockchain technology and the Internet of Things (IoT) has grown rapidly due to an increase in media coverage. Many different blockchain applications and platforms have been developed for different purposes, such as food safety monitoring, cryptocurrency exchange, and secure medical data sharing. However, blockchain platforms cannot store all the generated data. Therefore, they are supported with data warehouses, which in turn is called a hybrid blockchain platform. While several systems have been developed based on this idea, a current state-of-the-art systematic overview on the use of hybrid blockchain platforms is lacking. Therefore, a systematic literature review (SLR) study has been carried out by us to investigate the motivations for adopting them, the domains at which they were used, the adopted technologies that made this integration effective, and, finally, the challenges and possible solutions. This study shows that security, transparency, and efficiency are the top three motivations for adopting these platforms. The energy, agriculture, health, construction, manufacturing, and supply chain domains are the top domains. The most adopted technologies are cloud computing, fog computing, telecommunications, and edge computing. While there are several benefits of using hybrid blockchains, there are also several challenges reported in this study.

## 1. Introduction

For the last few years, the global demand for using Internet of Things (IoT) devices is highly increasing due to the increasing global market demand for faster and more efficient ways of manufacturing, required improvements of the military capabilities, and transforming things into smart ones such as smart homes, smart factories, and smart cities. Although IoT devices have numerous benefits, they also have several weaknesses, such as generating a huge amount of data, requiring a lot of energy to work, and considerations regarding the trust issues as they are centralized, controlled by an administrator who can manipulate the underlying system or even stop it entirely. The IoT system enables the devices to collect data about themselves and the environment around them, and later share these collected data with a device, and finally send these data to a central server. Blockchain technologies allow the IoT devices to exchange collected data with each other or send them to a cloud server securely and reliably [1]. As a result, blockchain technology has been introduced to minimize these potential weaknesses and risks.

Nakamoto [2], who is the pseudonym of the creator of Bitcoin, introduced the first cryptocurrency that uses distributed ledger technology (DLT) (a.k.a., blockchain). Since then, blockchain technology has penetrated the Internet of Things (IoT) market, allowing smart devices that can connect to the Internet to use a secure, immutable, and verifiable network. Blockchain is a decentralized ledger that secures, verifies, and records all peer-to-peer transactions quickly, safely, and transparently. The primary benefit of using blockchain technology over traditional technologies is that it enables two parties to perform secure transactions online without the need for a trusted authority. As a result of the lack of this authority, transaction rates are cheaper than the other conventional approaches [3].

As the world is becoming more and more dependent on smart devices, the number of connected IoT devices by the year 2025 is estimated to be 16.44 billion devices, and 25.44 billion by the year 2030 [4]. As such, we expect a dramatic change in the IoT market, and the contribution of this new blockchain technology is expected to be disruptive. Many vendors are currently developing new platforms, tools, and techniques. While blockchain platforms are very useful in terms of security and transparency, all the generated data cannot be stored in these platforms. In most cases, a separate data warehouse is needed to store the huge amount of data that cannot be stored directly in the blockchain platform. This can be a cloud data warehouse or a traditional central database management system; however, cloud data warehouses are mostly preferred due to their elasticity and other advanced features. Many blockchain applications and platforms have been developed recently using the cloud as storage units.

Since blockchain platforms cannot store all the generated data, they are mostly supported with cloud data warehouses, which can be called a hybrid blockchain platform. Another perspective for hybrid blockchain definitions is the use of both public and private blockchains in the same project. Ref. [5] described the hybrid blockchain as a street with many stores, where everyone can access and view the stores, similar to public blockchains, however, one cannot access the back offices of the stores without permission, which is similar to the private blockchain. From this point of view, a hybrid blockchain can be considered as a combination of a private and a public blockchain where the private blockchain can be hosted on the public blockchain. A hybrid blockchain can be entirely customized, where hybrid blockchain users can decide which transactions are made public or who can take part within the blockchain.

While several systems have been developed based on the idea of hybrid blockchains, a systematic overview of the current state of the art on the use of hybrid blockchain platforms is lacking. Knowing how this integration has been performed would help facilitate future research on hybrid blockchains. Although there are several relevant papers on this topic, this has not been evaluated in detail yet. The objective of this study is to present the main challenges and possible solutions and, also, different aspects related to this hybrid blockchain research. As such, we performed a systematic literature review study to collect and synthesize the required data on the state-of-the-art in this field.

In this paper, we particularly focus on the integration of blockchain and IoT, its motivations, challenges, and the domains by performing a systematic literature review (SLR) on research articles collected from different digital databases.

The following research questions are defined in this SLR study:

1. What are the key motivations for adopting hybrid blockchain?
2. What kind of domains has this concept been applied to?
3. What are the adopted technologies in IoT and blockchain integration?
4. What are the blockchain platforms used in the IoT and blockchain integration?
5. What are the key challenges and possible solutions for IoT and blockchain integration?

The contributions of this study are as follows:

- To the best of our knowledge, this is the first systematic review of the hybrid blockchains in literature.
- We evaluated 38 research papers (see Appendix A) from different dimensions and responded using different categories for each research question.
- Challenges and possible solutions are also discussed in this paper; this might pave the way for further research.

This first SLR study using 38 research articles on hybrid blockchains shows that efficiency, data integrity, and security are the major motivations for adopting integration of IoT and blockchains. Researchers mostly focused on health, energy, agriculture, and manufacturing domains and applied fog computing, edge computing, telecommunications, and cloud computing technologies. The most preferred blockchain platform is Ethereum, and several challenges are discussed in this study. The following sections are organized as follows: Section 2 provides the background and related work. Section 3 describes the adopted research methodology. Section 4 presents the results of this SLR, and Section 5 presents the discussion. Finally, Section 6 discusses the conclusions and future work.

## 2. Background and Related Work

The blockchain-integrated IoT system (BC-IoT system) can be defined as an IoT system that contains some blockchain elements to perform its transactions. Therefore, understanding the architecture of the IoT systems and the structures and operations of blockchain networks is necessary for the analysis of BC-IoT systems. In this section, we provide an overview of the background information. We also present some related studies in this section.

### 2.1. Background

#### 2.1.1. Internet of Things

The Internet of Things (IoT) refers to a set of devices that are connected to the Internet or other communication networks and exchange data among themselves. Any object can be transformed into an IoT device by adding sensors and processing ability. For instance, very large and crowded cities can also be covered with thousands of tiny IoT components to track the traffic, and useful suggestions and proper measures can be provided to eliminate several problems. It seems possible to turn anything into an IoT device thanks to the availability of very cheap and tiny computer chips, and the widespread use of wireless networks. Along with using IoT devices to make daily life easier, IoT can also be used in different application domains shown as follows:

1. Manufacturing [6]: Due to the increasing population numbers in the last few decades, the demand for goods is as never before. IoT devices are being adopted in today’s manufacturing to automate production lines, which highly increase the production speed and, thereby, reduce the overall costs. Less labor is needed to produce the same amount of goods and, therefore, manufacturers need to pay less money for the labor.
2. Healthcare [7]: Medical IoT devices are being used as remote patient management (RPM) tools by physicians to monitor the medical state of a patient, distantly. IoT devices can be wearable or implantable devices, and they can help medical doctors to monitor heartbeat, arrhythmia, blood pressure, oxygen level, sugar level, and they can even be used for collapse detection.
3. Environment [8]: Smart sensors can help to fight against climate change and make the world greener as IoT devices are also used to measure CO_2_ levels, oxygen levels, and ozone concentration in the atmosphere. They can monitor volcanic activities, extreme weather conditions, water levels, and safety-related events, and help to predict the timing of occurrences of natural disasters such as earthquakes, tsunamis, and wildfires.
4. Energy [9]: Energy waste is another problem that IoT is used to prevent. Sensors are used to sense and transmit real-time data regarding the energy levels being produced and consumed. They can be used to track the sunlight and direct the solar panels to the appropriate positions to maximize performance.
5. Agriculture and our food supply [10]: In precision agriculture, IoT sensors are widely used; for example, in smart greenhouses they are used to monitor and control temperature and humidity to increase yield [11]. In addition, some apps can advise farmers what time is the best to transplant their crops and harvest them.

IoT systems mainly consist of the following subsystems: perception layer, communication layer, and industrial applications. The perception layer is the physical layer of the IoT system, where sensors, RFID tags, barcode or QR code readers, and other data-collecting devices are used to collect data. After these data are collected, the communication layer connects the IoT device with a gateway device such as Wi-Fi access points (APs) using a communication protocol (e.g., Bluetooth, NFC, and Ethernet). The communication layer transfers the collected data to the industrial applications layer where data are being analyzed and stored.

#### 2.1.2. Blockchain

Blockchain (BC) is a decentralized ledger that securely, verifiably, and transparently records all transactions made on the blockchain network. The ledger is shared among distributed computers (a.k.a., nodes) on the network. All users can see the ledger from its first transaction in the system until its most recent one as it is not controlled or owned by a central entity, being decentralized. When a user sends a transaction, the data of the transaction are encrypted using a cryptographic algorithm before being verified by the miners to check if the transaction is valid. If most of the miners consent to the transaction, a new block is added to the chain [12]. The primary benefit of blockchain over traditional technology is that it allows two parties to conduct encrypted transactions over the Internet without the intervention of a third-party entity.

Blockchain technology was proposed to support transactions between two parties in a peer-to-peer manner without the need for a middleman using a cryptocurrency called Bitcoin. This initial blockchain technology was then labeled as blockchain 1.0. Later, new blockchain technology emerged that allows applications to be built on top of the blockchain platform, and smart contracts were widely used. The use of such smart contracts helped to realize decentralized applications (Dapps), decentralized autonomous organizations (DAOs), smart land, smart tokens, and other cryptocurrencies that allowed the capability for automated financial applications. These applications in the financial sector were developed using smart contracts, which are now called blockchain 2.0. However, blockchains are not only restricted to cryptocurrency, which is just one application of the wider definition of DLT.

Distributed ledgers can store arbitrary data that are not always linked to financial services. All implementations of blockchain technology that include a broader range of non-cryptocurrency-distributed ledger uses are called blockchain 3.0 [13]. Blockchain technology consists of the following four main components: a smart contract, consensus, ledger, and cryptography [14]. The smart contract is a kind of program stored on the blockchain that starts functioning when the terms of the contract are achieved. The consensus is an agreement that all nodes of the blockchain follow to determine which information is added to the next block of the ledger and provide validity and authenticity for the transactions on the blockchain. There are two main categories of consensus listed as follows:

6. PoW (proof of work) [15]: This consensus mechanism is used by Bitcoin [16], Ethereum 1.0 [17]. All nodes are a part of a competition. In this competition, each node tries to construct the appropriate block by solving a mathematical puzzle, which is called mining. The transaction fees in this consensus are calculated based on the demand and supply of transactions, where miners will choose to verify transactions with the highest fees first when the number of waiting transactions exceeds the number that one block of the blockchain can contain, which is why Eth 1.0’s transaction fees are so high sometimes. However, the problem with PoW for a blockchain is that it is very expensive as it requires a huge amount of computational power to mine; therefore, if the awarded coins drop in price and becomes cheaper than the energy costs spent, then miners will have no incentive to mine more blocks of that blockchain.
7. PoS (proof of stake) [15]: Unlike the PoW, PoS does not require high computational power to validate block transactions. The more coins a miner has, the more mining rewards and power over the network they have. This consensus mechanism is significantly cheaper than PoW, and its transaction fees are very low. Some examples of blockchains using PoS are Eth 2.0 [18], Cardano [19], Solana [20], and Polkadot [21].
8. Other consensus mechanisms, such as delegated proof of stake [22], practical Byzantine fault tolerance [23], proof of elapsed time [24], practical Byzantine fault tolerance [25], proof of weight [19], proof of burn [24], proof of capacity [26], and proof of space [27], also exist; however, they are not as widely used as PoW and PoS.

The ledger is a database that contains all the transactions that occurred in the blockchain. Since the network is decentralized and there is no central authority, the ledger is distributed across the network. Every transaction added to the ledger can never be deleted, which makes the ledger immutable. In addition, to make sure that all the information on the blockchain network is accessed by only authorized users, cryptography is used. Since the blockchain is a decentralized network and there are no centralized entities that control and store the transactions of the network, a P2P network is used when a sender wants to make a transaction. When a sending wallet wants to make a transaction, it uses a public and a private key. The public key is used as an identifier of the sending wallet in the network and the private key is used to sign the transactions of the wallet in the network to protect the authenticity and integrity of the transaction on the network. After the transaction is signed with the private key, the wallet broadcasts the request to all the nodes on the network of the blockchain, where all the nodes verify all the transactions of the blockchain and start to validate the transaction and check if the request is not tempered. Once the request is successfully validated by more than 50% of the nodes on the network, a new block is added to the last block on the blockchain, where each block contains various such validated transactions with a timestamp, hash, and the hash of the current block.

Hybrid blockchain platforms are used to integrate IoT systems with the blockchain; some projects that use this integration have different architecture types. Ref. [28] proposed a hybrid–IoT system that uses multiple PoW blockchains as sub-blockchains for IoT, where hundreds of IoT devices located at a near distance from each other are contained in a sub-blockchain. A Byzantine fault-tolerant interconnector is used to ensure the transactions are between the sub-blockchains. Ref. [29] proposed a hybrid blockchain as a crowdsourcing platform and used a public chain and many private sub-chains. It uses delegated proof of stake (DPOS) and practical Byzantine fault tolerance (PBFT) consensuses to verify the transactions.

### 2.2. Related Work

During our search in electronic databases, there was no other SLR paper that focused on hybrid blockchains. A paper that focused on making the Internet and IoT more secure by using blockchain smart contracts is the study of Lone and Naaz [30]. Their paper examines the applicability of blockchain smart contracts to achieve the security goals related to the Internet and, particularly, IoT. While their paper defined four research questions, our SLR paper focuses on five research questions. There is one similar question, which is related to the blockchain platforms. Similar to our results, they also specified that the Ethereum platform is the most exploited platform in the selected papers. They concluded that access control, authentication, integrity assurance, data protection, secure key management, and nonrepudiation are the most common smart contract-driven security services in the Internet and IoT. Ref. [31] focused on how the blockchain and smart contracts work with IoT. They reported that the blockchain that combines blockchain and IoT can be very powerful. A smart contract allows the automation of the complex multistep process. They also concluded that if IoT devices in an IoT ecosystem are combined to work together, they can automate time-consuming workflows and achieve cryptographic verifiability by reducing cost and time. Ref. [32] studied the blockchain architectures that governments use in public services, where they focused on the software architectures and solutions of blockchain technology applied in public services. Their research results conclude that the blockchain solutions are diversified and the offered solutions are developed recently, which opens the road for more research in the future. Ref. [33] studied the maturity and readiness of digital forensic (DF) investigations in the era of the industrial revolution (IR) 4.0, where they focused on the challenges that face DF in the IR 4.0, the readiness, the existing maturity model, and benchmarking the maturity element. They were able to outline five indicators that need to be considered to support the DF organization’s maturity model related to IR 4.0. They were also able to list out 28 suggested governance and management objectives that DF organizations can use to guide them concerning IR 4.0.

Tran et al.’s study [2], on the other hand, is the most relevant paper to this SLR. This paper focused on the ways to integrate blockchain with IoT and how to achieve this integration. The paper reported that security, integrity, reliability, and performance are the most common objective reasons for adopting the integration; another interesting reason for the integration is to add new functionalities to the IoT systems. Problem-wise reasons for the adoption are to decentralize operations and improve the security of IoT systems. Most of the reviewed BC-IoT systems are integrated with one blockchain network only, and the most common blockchain network is Ethereum. The business process orchestrator, authorization mechanism, and sensor data storage are the top three modules added to the IoT systems by the blockchain networks. Most of the verified transactions recorded on the blockchain are resource exchanges and interactions with devices and services data.

## 3. Research Methodology

**Figure 1:**
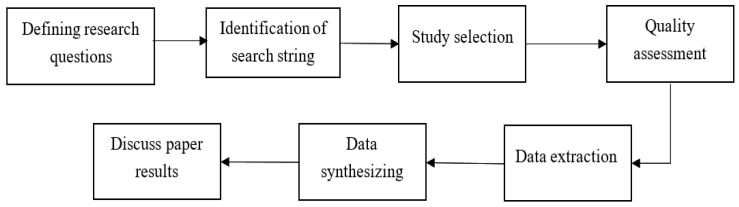
SLR process.

To achieve the objective of answering the research questions, this SLR paper has been prepared by following the guidelines provided by [34]. The following three stages are followed: planning, conducting, and reporting the systematic literature review. In Figure 1, the process of conducting this SLR is depicted. This process was followed, and results were gathered.

### 3.1. Research Questions

This research’s goal is to analyze published studies and their findings on the integration of the blockchain and IoT. To make the paper more focused, Table 1 shows the six research questions we developed.

### 3.2. Primary Research Questions

To find the primary studies needed for this SLR paper, we used the following digital databases: ScienceDirect (www.sciencedirect.com, accessed on 5 October 2021), ACM Digital (dl.acm.org, accessed on 5 October 2021), IEEE Explore (ieeexplore.ieee.org, accessed on 5 October 2021), and Wiley (www.wiley.com, accessed on 5 October 2021). This set was selected because these are the databases that index the most important conferences and journals in the computer science discipline. Later, a search criterion was set as follows: (“Blockchain”) AND (“Internet of Things”) AND (“Architecture” OR “Integration” OR “Cloud”).

The search resulted in a total number of 985 research articles. A total of 804 of them were found in the IEEE Xplore database, 118 in ScienceDirect, 38 in ACM Digital, and 25 in the Wiley database. We eliminated any review articles, correspondence articles, and discussion papers. This filter reduced the studies to 295 articles, where the results found in IEEE Xplore were reduced to 175, papers in ScienceDirect to 75, papers in ACM Digital to 29, and papers in Wiley to 16. Later, exclusion criteria were applied to exclude irrelevant publications. The relevant ones were added to a spreadsheet file. The exclusion criteria (EC) are provided in Table 2.

**Figure 2:**
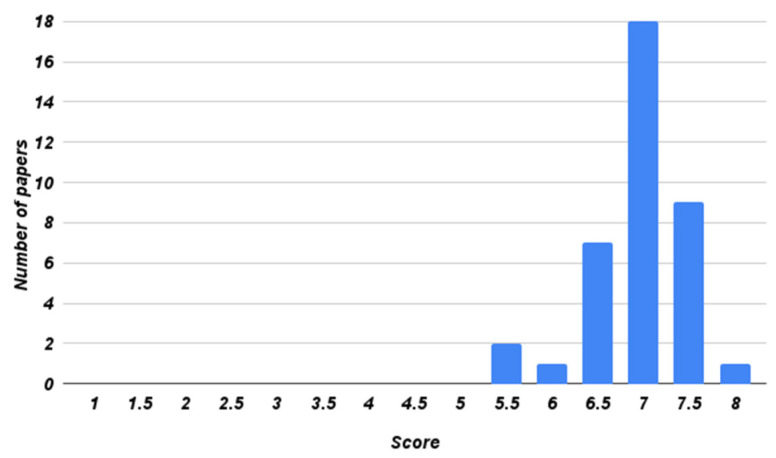
Distribution of the selected papers’ quality score.

The selected publications were then checked using quality assessment questions to ensure that only high-quality publications were being used. Each question was assessed with a score of 1 (yes), 0 (no), or 0.5 (partial). Therefore, 0 is the minimum score and 8 is the maximum score for a paper. A paper with a total score of 4 or lower was excluded. Eight assessment questions were used from the study of [35] because this set of questions is widely used in SLR papers. The assessment questions that we used are shown in Table 3. Figure 2 shows the distribution of the selected papers’ quality scores.

After the quality assessment was performed, 38 publications were identified for the SLR study. Therefore, observations and conclusions presented in this study are based on these 38 publications. Figure 2 shows that most of the papers achieved high scores to provide higher quality.

### 3.3. Data Extraction

After selecting the papers, data relevant to the research questions were extracted, stored, and categorized in a spreadsheet. The data extraction form, which contains the essential data needed for this study, is shown in Table 4. Papers were read in full and required data were collected. The collected data per question were then categorized into different groups. In RQ1, the motivations were categorized into the following groups: security, transparency and trust, efficiency, privacy, and quality of service. In RQ2, the domains were categorized as follows: energy, agriculture, health, construction, manufacturing and supply chain, automotive and transportations, education, military, and government. In RQ3, the adopted technologies were categorized into the following categories: cloud computing, fog computing, telecommunications, edge computing, and extended reality. In RQ4, the BC platforms were categorized into the following categories: Ethereum, Bitcoin, Litecoin, EOS, and Ripple. In RQ5, the challenges were categorized into the following categories: security and privacy, storage and scalability, computational power, bandwidth and connectivity, and cost. In addition to these essential elements, general data, such as the title and publication year, were also collected. Table 4 shows the collected elements.

### 3.4. Data Synthesis and Reporting

After we managed to extract and categorize the data, the aggregated data were then synthesized to be used to respond to research questions.

## 4. Results

**Figure 3:**
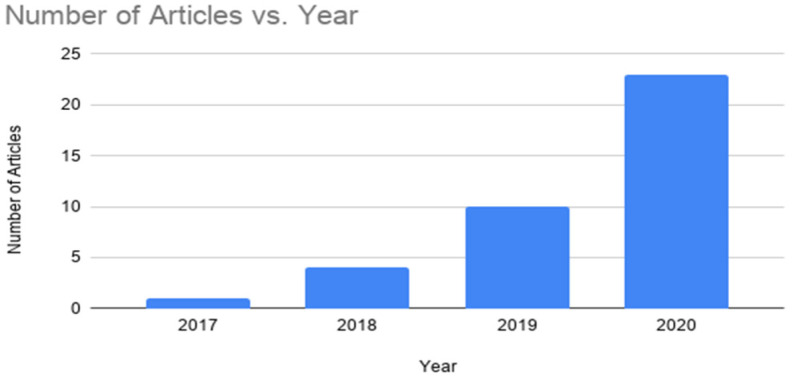
Number of papers per year.

In this section, the results of this systematic literature review are presented. The number of selected papers for the last years is presented in Figure 3. A clear increasing interest in the recent years can be seen from that figure. In Table 5, the number of papers that are published in different databases is shown, where ScienceDirect is the primary, and IEEE Explore is the secondary, source.

The six research questions presented in Table 1 are addressed one by one in the following subsections:

1. RQ-1: What are the key motivations for adopting hybrid blockchain?

**Figure 4:**
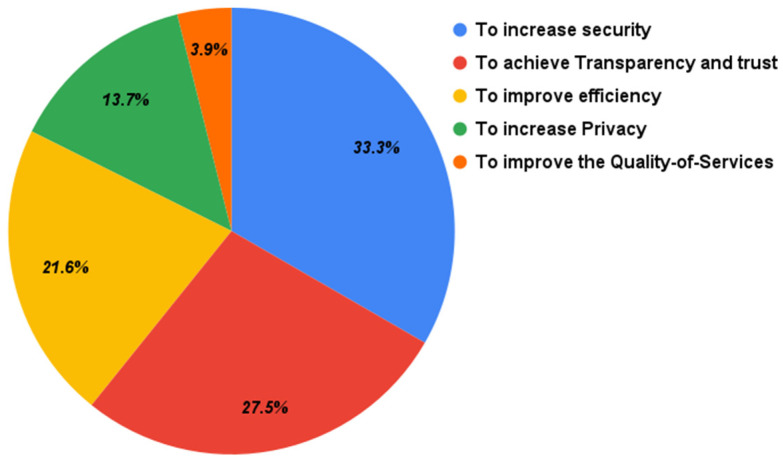
Motivations of adopting a hybrid blockchain.

The motivations identified from the primary studies are shown in Figure 4. The results show that more than one-third of the primary papers had a motivation to increase security. Some of them were needed to ensure the integrity of the data collected by the IoT devices of the system [36,37,38,39] or to protect the confidentiality of the collected data [38,40,41], or to ensure the availability of the IoT systems [42] because there are no centralized authorities that can be attacked to stop the systems from functioning. In addition, another use case of blockchain as a security measure was to protect data from plaintext and ciphertext attacks on UAVs [43]. Another motivation was related to the transparency and trust goals, as the platform is resistant to the modification of the blockchain blocks. As a result, the data inside each block are unmodifiable and cannot be edited or deleted, which provides trust in the system. It can also be beneficial to track and trace products and increase the credibility of food safety information [44]. In addition, its distributed nature helps to increase transparency as all the stored data on the blockchain are accessible to everyone [38,43,45,46,47,48,49]. Efficiency is also an important motivation for the integration, as smart contracts can be used to reduce the delay between IoT devices [50], or to reduce costs [42,48,49,51], or to increase energy efficiency [24], or to decrease latency [48,52,53], or to enhance throughput [52]. Another important motivation of the integration was privacy [36,38,50,54]. A sender and a receiver are only known by their public keys, which do not provide any personal data.

2. RQ-2: What kind of domains has the hybrid blockchain been applied to?

**Figure 5:**
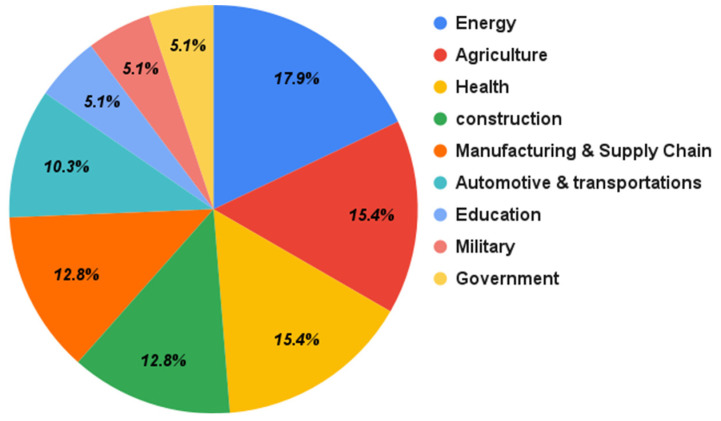
Hybrid blockchain domains that have been adopted.

Figure 5 shows the percentage of domains that adopted hybrid blockchains. As shown in Figure 5, energy is the most mentioned domain in the primary papers, with 17.95% of the papers. Agriculture and health are second and third, with 15.38%. These results indicate that these three domains are the most adopting domains of the integration. Other domains were construction and manufacturing and supply chain domains with 12.82%, automotive and transportations (10.26%), and education, military, and government, with 5.13%.

3. RQ-3: What are the adopted technologies in the IoT and blockchain integration?

**Figure 6:**
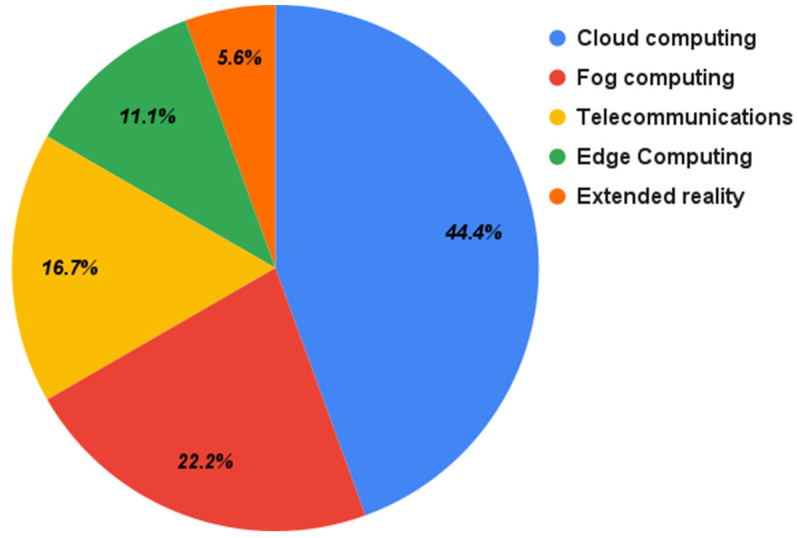
Adopted technologies in the integration.

Figure 6 shows the distribution of technologies used in these selected papers. Cloud computing is the most adopted technology, with 44.4%. It includes cloud storage and cloud servers. Fog computing is the second most adopted technology with 22.2%, followed by telecommunications with 16.7%, edge computing with 11.1%, and extended reality with 5.6%. Extended reality includes both virtual reality (VR) and augmented reality (AR) technologies.

4. RQ-4: What are the blockchain platforms used in the IoT and blockchain integration?

**Figure 7:**
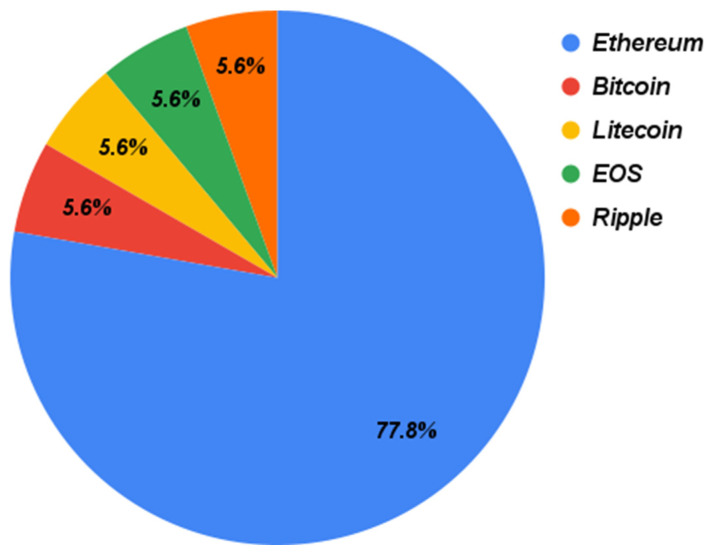
The adopted BC platforms in the primary papers.

Figure 7 shows the blockchain platforms used in the selected papers. According to this figure, Ethereum is the top-used blockchain platform with 77.8%, as Ethereum is considered a mature blockchain technology for developing smart contracts [37,51]. EOS blockchain is another platform that was also used, as its smart contract platform enables IoT to be integrated with the blockchain [55]. Bitcoin, Litecoin, and Ripple were also used in these papers. Ref. [36] stated that Bitcoin and Litecoin can be used as a medium to store the IoT data. Ripple, on the other hand, was used as a private blockchain to establish private communications between nodes [56]. Bitcoin, Litecoin, EOS, and Ripple have been used, with 5.6% in the selected studies.

5. RQ-5: What are the key challenges and possible solutions of IoT and blockchain integration?

We categorized the challenges into five categories. Table 6 presents these categories and possible solutions. These five categories are described as follows:

6. Portability: It is almost impossible to enable blockchain’s required features in most modern industrial machines because the protocols that are being used in the blockchain operations and transactions are very specific while being computationally intense, thread-blocking, and time-consuming. These issues can be solved by designing a system that can decouple the operations of the blockchain from industrial machines’ functionalities and capabilities [37].
7. Resource: Replacing currently functional legacy systems with blockchain will cost time and resources, but it can be resolved by creating a mechanism that enables the communication of the blockchain and the legacy systems rather than replacing it with a fully decentralized system [57].
8. Interoperability: Industrial IoT devices are heterogeneous. Old and new devices use different operating systems, of which some are very difficult to modify to add the blockchain features. To solve this issue, an abstraction layer in the software architecture design of the OS can be added to allow the communication of the IoT devices with the smart contracts of different blockchains [37].
9. Computational power: The use of the PoW consensus mechanism requires high computational power to mine new blocks on the blockchain. This requirement costs a lot of money and too much electrical power. Ref. [47] propose a solution as a gateway node that can be used to gather the blocks of data from a set number of IoT devices and then verify the blocks as a miner before it adds them to the blockchain network.
10. Scalability: Technical limitations of traditional blockchains cannot scale well for widespread use in an IoT environment. Ref. [52] proposed the use of “off-chain” protocols, where some of the transactions are moved temporarily to be computed elsewhere and then return the results of the transactions to be added to the main chain.

The scalability limitations of blockchain networks are a big obstacle for blockchain applications to perform large-scale transactions. Ref. [58] proposed a blockchain and bilinear mapping-based data integrity scheme (BB-DIS) for high-scale IoT data in cloud storage as a solution to this challenge.

## 5. Discussion and Threats to Validity

In Section 5.1, a general discussion addressing research questions is presented. In Section 5.2, potential threats to validity are explained. In Section 5.3, the specialty of hybrid blockchains in the IoT environment compared to general hybrid blockchains is discussed. In Section 5.4, several research directions are suggested.

### 5.1. Discussion

In this paper, we reviewed the literature on the integration of blockchain platforms and IoT to understand the state-of-the-art and current practices. For this purpose, five research questions were identified and responded to. RQ1 aimed at understanding the key motivations for adopting the hybrid blockchain with IoT. Security, transparency, trust, and privacy were the top motivations. This shows that most of the research groups had mostly security-related concerns and therefore, adopted this new strategy. RQ2 explored the domains where the integration has been applied. Energy, agriculture, health, and construction were the top domains. The energy sector showed the power of blockchains earlier than the other sector and, therefore, we noticed that this type of hybrid blockchains was mostly used in the energy domain. Some other domains were not mentioned in the articles, which are the entertainment and business domains. These two domains are witnessing a major development and adoption with the hybrid blockchain that could change the way people interact at their work, play video games, or attend concerts. RQ3 focused on the technologies that were used in this integration. Cloud computing, fog computing, and communications were the top results. Since the IoT devices and sensors are a major part of the blockchain and IoT integration, they were not considered as a technology, but rather as a part of the system. As shown in these results, cloud computing plays a major role in this integration because the generated huge amount of data is mostly stored in cloud computing platforms. RQ4 addressed which blockchain platforms were used. During our analysis, Ethereum was used with 77.8%, followed by Bitcoin, Litecoin, EOS, and Ripple. This indicates that the majority of the projects are relying on Ethereum. Therefore, any attack or network failure on the Ethereum blockchain can cause operational failures in these systems. RQ5 identified the key challenges and possible solutions faced by prior researchers. The collected challenges were mainly the challenges of integrating the blockchain and IoT systems. Challenges were reported based on the explicit statements in the articles. There can be more challenges; however, if they were not mentioned in these papers, we could not identify and include them here. The integration of blockchain technology and IoT is still in its early stages and yet being widely adopted in various domains and sectors.

### 5.2. Threats to Validity

We can see new domains and new technologies soon that will emerge as a result of this integration. There are several threats to validity in this SLR. Concerning the timeframe, the primary papers selection process was finalized in October 2020. This SLR selected the papers that were published until that time. Papers that were published on the digital databases after this month were not considered in this review. Because of the fast development of the blockchain and IoT space, there may be new papers that have not been covered in this SLR. Another threat to validity is selecting the articles. Different papers could be found when different databases were used for the primary paper selection. However, we did not want to use Google Scholar because it indexes non-peer-reviewed papers and non-well-reputed journals as well. Moreover, during the data extraction process, some data might have been missed, and to reduce this threat, the authors double-checked all primary papers. In addition, the search for the primary papers was strictly focused on papers in English; as such, there could be a chance of missing some papers that were written in other languages that could add value to the research questions in this paper. Some papers used the term hybrid blockchain, however, their definition was different than our scope. For example, one of these papers referred to the combination of public and private blockchains [59]; however, since IoT was not included in this integration, it was not used in the analysis. In addition, papers that focused on only blockchains were not included in the SLR analysis [60].

### 5.3. Specialty of Hybrid Blockchains in IoT Environment compared to General Hybrid Blockchains

There are specific requirements needed for hybrid blockchains in IoT environments compared to general hybrid blockchains. One of the most important issues is the resource limitations of IoT devices [61]. The platform should not cause an extra bottleneck on the devices. In addition, the scalability of hybrid blockchain platforms in the IoT context is crucial, and therefore, microservices were applied in one of the studies to address this requirement [61]. Confidentiality is another quality factor that needs to be addressed for hybrid platforms in IoT environments because data produced from different devices such as smart home appliances and wearables are sensitive and confidential [61]. For general hybrid blockchains, scalability and confidentiality have less impact on the design of the overall hybrid blockchain architecture. Throughput is another parameter that requires extra design decisions during the system design because IoT applications need a huge number of transactions to be executed at a time, however, some of the blockchain platforms such as Bitcoin cannot satisfy the expectations (e.g., only seven transactions per second) because of their internal design [61]. Latency can be mostly tolerated in hybrid blockchains in the IoT context and it is known that latency is high in some blockchain platforms such as Bitcoin (i.e., 10 min to complete a transaction). Maintaining hybrid blockchain in an IoT environment is more costly because the required computational power, energy, and storage are much more. These different quality aspects make hybrid blockchains in the IoT context more special compared to the general hybrid blockchains.

### 5.4. Research Directions

As part of this SLR study, we identified the following research directions:

1. Artificial Intelligence (AI)-enabled Hybrid Blockchains: Machine learning algorithms, and more specifically, deep learning algorithms have been applied in many different application domains successfully recently. In the cloud data warehouse, these algorithms can be effectively used, and interesting patterns can be discovered. However, the learning types (i.e., supervised, unsupervised, semisupervised, reinforcement learning) and corresponding algorithms (e.g., support vector machines, K-means clustering, low-density separation, Deep Q Network) must be carefully selected. From an engineering perspective, the integration of machine learning capabilities into the hybrid blockchain requires additional research in this field. The isolated development of these AI components limits their benefits and, therefore, the system engineering perspective must be followed.
2. Energy-Efficient Hybrid Blockchains: Energy efficiency is one of the most important concerns of blockchain platforms. Some decentralized consensus mechanisms such as proof-of-stake (PoS) are more efficient than others, such as the proof-of-work (PoW) model. However, they are still not considered to be energy-efficient, and more research is needed to optimize the hybrid blockchains in IoT environments. New consensus protocols in this context can reduce the required resources. For example, recently a new blockchain network called Casper demonstrated that it is 47,000% and 136,000% more energy-efficient than Ethereum and Bitcoin platforms, respectively [62]. Energy efficiency is not necessarily related to only the consensus mechanism; there are other aspects that need to be investigated in detail in future research.
3. Interoperable Hybrid Blockchains: Between two or more hybrid blockchains in the IoT context, there should be an effective communication mechanism to obtain more benefits and achieve more transparency and easier processes. While there are some solutions at the blockchain level, more research is needed for complex hybrid blockchains.
4. Ethical and Legal Aspects: Legal boundaries of restrictions and ethical aspects must be investigated in hybrid blockchains, which are used by a consortium. Ethics and moral issues of hybrid blockchains are also crucial, but now they are lacking.
5. Privacy-preserving Hybrid Blockchains: Privacy preservation for hybrid blockchains in IoT environments is another important issue that needs further research because sensitive and confidential data are stored on some platforms. Since most of these systems are public and transactions are visible to other network members, confidential information might be inferred by adversaries. Therefore, new privacy preservation strategies are needed.
6. Standardization: In the IoT context, one of the most important challenges is standardization. While there are different initiatives at the national and international levels, there is still no standard set because the IoT standards landscape is too diverse. In the long term, standardization should be also managed for hybrid blockchains in the IoT environments.

## 6. Conclusions and Future Work

In this SLR paper, 38 papers were used as primary papers, and five research questions were addressed. Security, data integrity, and efficiency are the top three motivations for adopting integration. The energy, agriculture, health, construction, manufacturing, and supply chain domains are the top domains that adopt the integration. The most adopting technologies are cloud computing, telecommunications, fog computing, and edge computing. Ethereum was by far the most used blockchain in the reviewed articles. The reported challenges are related to portability, resources, interoperability, computational power, and scalability. As future work, we are planning to design and implement a hybrid blockchain platform that can minimize the reported challenges.

## Figures and Tables

**Table 1:** Research questions (RQs).

ID	Research Question (RQ)
Q1	What are the key motivations for adopting hybrid blockchain?
Q2	What kind of domains has it been applied to?
Q3	What are the adopted technologies in IoT and blockchain integration?
Q4	What are the blockchain platforms used in the IoT and blockchain integration?
Q5	What are the key challenges and possible solutions of IoT and blockchain integration?

**Table 2:** Exclusion criteria.

No.	Criterion
EC1	Not related to blockchain and IoT integration
EC2	Non-English publication
EC3	A survey or a review publication
EC4	Duplicated publication
EC5	The publication is older than 2017

**Table 3:** Quality assessment questions [35].

No.	Assessment Questions
Q1	Are the aims of the study clearly stated?
Q2	Are the scope and context of the study clearly defined?
Q3	Is the proposed solution clearly explained and validated by an empirical study?
Q4	Are the variables used in the study likely to be valid and reliable?
Q5	Is the research process documented adequately?
Q6	Are all study questions answered?
Q7	Are the negative findings presented?
Q8	Are the main findings stated clearly in terms of creditability, validity, and reliability?

**Table 4:** The data extraction form.

No.	Extraction Elements
1	ID
2	Title
3	Link
4	Year
5	Database
6	Publication channel
7	Type
8	Motivations
9	Domains
10	Adopted technologies
11	Blockchain platforms
12	Challenges and possible solutions

**Table 5:** Paper distributions per journal.

Data Sources	# of Papers
ScienceDirect	24
ACM Digital	4
IEEE Xplore	10
Wiley	0

**Table 6:** Challenges and possible solutions for BC and IoT integration.

Category	Challenges (C1 to C6)	Proposed Solutions (S1 to S6)	Reference
Portability	It is almost impossible to modify the industrial apparatus software to add the blockchain protocols.	To design a system that can decouple the operations of the blockchain from industrial machines’ functionalities and capabilities.	[37]
Resources	Replacing legacy systems with blockchain requires time and resources.	Creating a mechanism that enables the communication of the blockchain and the legacy systems rather than replacing it with a fully decentralized system.	[57]
Interoperability	Some operating systems (OS) of old IoT devices cannot be modified to add the new blockchain features.	Adding an abstraction layer in the software architecture design of the OS to allow the communication of the IoT device with the smart contracts of different blockchains.	[37]
Computational power	High computational power is required by IoT devices that use the PoW consensus mechanism.	A gateway node can be used to gather the blocks of data from a set number of IoT devices and then verify the blocks as a miner before it adds them to the blockchain network.	[47]
Scalability	Technical limitations of traditional blockchains cannot scale them for widespread use in IoT environments.	An “off-chain” protocol can be used, where some of the transactions are moved temporarily to be computed elsewhere and then return the results of the transactions to be added to the main chain.	[52]
	The scalability limitations of blockchain networks prevent the blockchain applications from performing high scale IoT data.	A BB-DIS system can be used to overcome the high-scale IoT data issues in cloud storage.	[58]

## Data Availability

Not applicable.

## Appendix A. Primary Studies (Sources Reviewed in the SLR)

1. Abou-Nassar, E. M., Iliyasu, A. M., El-Kafrawy, P. M., Song, O. Y., Bashir, A. K., & Abd El-Latif, A. A. (2020). DITrust chain: towards blockchain-based trust models for sustainable healthcare IoT systems. *IEEE Access*, *8*, 111223–111238.
2. Ch, R., Srivastava, G., Gadekallu, T. R., Maddikunta, P. K. R., & Bhattacharya, S. (2020). Security and privacy of UAV data using blockchain technology. *Journal of Information Security and Applications*, *55*, 102670.
3. Fan, K., Bao, Z., Liu, M., Vasilakos, A. V., & Shi, W. (2020). Dredas: Decentralized, reliable and efficient remote outsourced data auditing scheme with blockchain smart contract for industrial IoT. *Future Generation Computer Systems*, *110*, 665-674.
4. Fernández-Caramés, T. M., & Fraga-Lamas, P. (2018). A Review on the Use of Blockchain for the Internet of Things. *Ieee Access*, *6*, 32979–33001.
5. Garg, N., Wazid, M., Das, A. K., Singh, D. P., Rodrigues, J. J., & Park, Y. (2020). BAKMP-IoMT: Design of blockchain enabled authenticated key management protocol for internet of medical things deployment. *IEEE Access*, *8*, 95956–95977.
6. Ge, C., Liu, Z., & Fang, L. (2020). A blockchain based decentralized data security mechanism for the Internet of Things. *Journal of Parallel and Distributed Computing*, *141*, 1–9.
7. Hang, L., Ullah, I., & Kim, D. H. (2020). A secure fish farm platform based on blockchain for agriculture data integrity. *Computers and Electronics in Agriculture*, *170*, 105251.
8. He, S., Tang, Q., & Wu, C. Q. (2018, November). Censorship resistant decentralized IoT management systems. In *Proceedings of the 15th EAI International Conference on Mobile and Ubiquitous Systems: Computing, Networking and Services* (pp. 454–459).
9. Iqbal, S., Malik, A. W., Rahman, A. U., & Noor, R. M. (2020). Blockchain-based reputation management for task offloading in micro-level vehicular fog network. *IEEE Access*, *8*, 52968–52980.
10. Jain, R., & Dogra, A. (2019, July). Solar Energy Distribution Using Blockchain and IoT Integration. In *Proceedings of the 2019 International Electronics Communication Conference* (pp. 118–123).
11. Jeong, J. W., Kim, B. Y., & Jang, J. W. (2018, April). Security and device control method for fog computer using blockchain. In *Proceedings of the 2018 International Conference on Information Science and System* (pp. 234–238).
12. Kochovski, P., Gec, S., Stankovski, V., Bajec, M., & Drobintsev, P. D. (2019). Trust management in a blockchain based fog computing platform with trustless smart oracles. *Future Generation Computer Systems*, *101*, 747–759.
13. Kumar, A., Krishnamurthi, R., Nayyar, A., Sharma, K., Grover, V., & Hossain, E. (2020). A novel smart healthcare design, simulation, and implementation using healthcare 4.0 processes. *IEEE Access*, *8*, 118433–118471.
14. Kumari, A., Gupta, R., Tanwar, S., & Kumar, N. (2020). Blockchain and AI amalgamation for energy cloud management: Challenges, solutions, and future directions. *Journal of Parallel and Distributed Computing*, *143*, 148–166.
15. Liu, Y., Lu, Q., Chen, S., Qu, Q., O’Connor, H., Choo, K. K. R., & Zhang, H. (2020). Capability-based IoT access control using blockchain. *Digital Communications and Networks*.
16. Lokshina, I. V., Greguš, M., & Thomas, W. L. (2019). Application of integrated building information modeling, IoT and blockchain technologies in system design of a smart building. *Procedia computer science*, *160*, 497–502.
17. Ma, M., Shi, G., & Li, F. (2019). Privacy-oriented blockchain-based distributed key management architecture for hierarchical access control in the IoT scenario. *IEEE Access*, *7*, 34045–34059.
18. Mazzei, D., Baldi, G., Fantoni, G., Montelisciani, G., Pitasi, A., Ricci, L., & Rizzello, L. (2020). A Blockchain Tokenizer for Industrial IOT trustless applications. *Future Generation Computer Systems*, *105*, 432–445.
19. Pal, K. (2020). Internet of things and blockchain technology in apparel manufacturing supply chain data management. *Procedia Computer Science*, *170*, 450–457.
20. Rahman, M. A., Rashid, M. M., Hossain, M. S., Hassanain, E., Alhamid, M. F., & Guizani, M. (2019). Blockchain and IoT-based cognitive edge framework for sharing economy services in a smart city. *IEEE Access*, *7*, 18611–18621.
21. Robert, J., Kubler, S., & Ghatpande, S. (2020). Enhanced Lightning Network (off-chain)-based micropayment in IoT ecosystems. *Future Generation Computer Systems*, *112*, 283–296.
22. Roy, D. G., Das, P., De, D., & Buyya, R. (2019). QoS-aware secure transaction framework for internet of things using blockchain mechanism. *Journal of Network and Computer Applications*, *144*, 59–78.
23. Rožman, N., Corn, M., Požrl, T., & Diaci, J. (2019). Distributed logistics platform based on Blockchain and IoT. *Procedia CIRP*, *81*, 826–831.
24. Saurabh, S., & Dey, K. (2021). Blockchain technology adoption, architecture, and sustainable agri-food supply chains. *Journal of Cleaner Production*, *284*, 124731.
25. Sharma, P. K., Chen, M. Y., & Park, J. H. (2017). A software defined fog node based distributed blockchain cloud architecture for IoT. *Ieee Access*, *6*, 115–124.
26. Singh, S. K., Jeong, Y. S., & Park, J. H. (2020). A deep learning-based IoT-oriented infrastructure for secure smart city. *Sustainable Cities and Society*, *60*, 102252.
27. Singh, S. K., Rathore, S., & Park, J. H. (2020). Blockiotintelligence: A blockchain-enabled intelligent IoT architecture with artificial intelligence. *Future Generation Computer Systems*, *110*, 721–743.
28. Sittón-Candanedo, I., Alonso, R. S., Corchado, J. M., Rodríguez-González, S., & Casado-Vara, R. (2019). A review of edge computing reference architectures and a new global edge proposal. *Future Generation Computer Systems*, *99*, 278–294.
29. Sok, K., Colin, J. N., & Po, K. (2018, December). Blockchain and Internet of Things Opportunities and Challenges. In *Proceedings of the Ninth International Symposium on Information and Communication Technology* (pp. 150–154).
30. Tian, Z., Yan, B., Guo, Q., Huang, J., & Du, Q. (2020). Feasibility of identity authentication for IoT based on Blockchain. *Procedia Computer Science*, *174*, 328–332.
31. Torky, M., & Hassanein, A. E. (2020). Integrating blockchain and the internet of things in precision agriculture: Analysis, opportunities, and challenges. *Computers and Electronics in Agriculture*, 105476.
32. Uddin, M. A., Stranieri, A., Gondal, I., & Balasubramanian, V. (2020). Blockchain leveraged decentralized IoT eHealth framework. *Internet of Things*, *9*, 100159.
33. Venkatesh, V. G., Kang, K., Wang, B., Zhong, R. Y., & Zhang, A. (2020). System architecture for blockchain based transparency of supply chain social sustainability. *Robotics and Computer-Integrated Manufacturing*, *63*, 101896.
34. Wang, H., & Zhang, J. (2019). Blockchain based data integrity verification for large-scale IoT data. *IEEE Access*, *7*, 164996–165006.
35. Xie, L., Ding, Y., Yang, H., & Wang, X. (2019). Blockchain-based secure and trustworthy Internet of Things in SDN-enabled 5G-VANETs. *IEEE Access*, *7*, 56656–56666.
36. Xu, H., Klaine, P. V., Onireti, O., Cao, B., Imran, M., & Zhang, L. (2020). Blockchain-enabled resource management and sharing for 6G communications. *Digital Communications and Networks*, *6*(3), 261–269.
37. Zhang, A., Zhong, R. Y., Farooque, M., Kang, K., & Venkatesh, V. G. (2020). Blockchain-based life cycle assessment: An implementation framework and system architecture. *Resources, Conservation and Recycling*, *152*, 104512.
38. Zhao, Q., Chen, S., Liu, Z., Baker, T., & Zhang, Y. (2020). Blockchain-based privacy-preserving remote data integrity checking scheme for IoT information systems. *Information Processing & Management*, *57*(6), 102355.
